# Association between Household Food Insecurity and Asthma in Korean Adults

**DOI:** 10.3390/ijerph16122115

**Published:** 2019-06-14

**Authors:** Seo-Hee Park, Byung-Jin Park, Dong-Hyuk Jung, Yu-Jin Kwon

**Affiliations:** 1 Department of Family Medicine, Yong-In Severance Hospital, Yonsei University College of Medicine, Yong-In 17046, Korea; SHEEPSH@yuhs.ac (S.-H.P.); BJPARK96@yuhs.ac (B.-J.P.); BALSAN2@yuhs.ac (D.-H.J.); 2 Department of Medicine, Graduate School of Yonsei University College of Medicine, Seoul 03722, Korea

**Keywords:** asthma, household food insecurity, Korean adults

## Abstract

Household food insecurity has been associated with noncommunicable diseases. The aim of this study was to investigate the association between household food insecurity and asthma in Korean adults. Household food security statuses were classified into three groups: Food-secure household, food-insecure household without hunger, and food-insecure household with hunger. The odds ratios and 95% confidence intervals for the presence of asthma according to household food security status were calculated using multiple logistic regression analyses after adjusting for confounding factors. A total of 14,770 participants were included in the analysis. The prevalence of asthma was 2.6% in those with a secure food status, 3.2% in those with an insecure food status without hunger, and 7.6% in those with an insecure food status with hunger (*p* < 0.001). Compared with that in participants with a household food secure status, the odds ratios (95% confidence intervals) for asthma were 1.12 (0.73–1.73) in those with a food-insecure household without hunger status and 2.44 (1.33–4.46) in those with a food-insecure household with hunger status after additionally adjusting for confounding factors. We found that household food insecurity with hunger was significantly associated with asthma prevalence in Korean adults. Implementation of household food security screening and public health intervention could be helpful to prevent and reduce asthma in adults.

## 1. Introduction

According to the 1996 World Food Summit, food security is defined as a state “when all people, at all times, have physical and economic access to sufficient, safe, and nutritious food to meet their dietary needs and food preferences for an active and healthy life” [1]. At the household level, food security involves the accessibility, sufficiency, security, and sustainability of food to meet the dietary needs of all members of the household [2]. Although the global economy is growing, approximately one out of every nine people worldwide is still undernourished [3]. A recent study of 134 countries reported that 10.8% of individuals in high-income countries and 56.5% in low-income countries live in conditions of food insecurity [4]. In Korea, a tool for assessing household food insecurity by classifying children and adults was first introduced in 2012 through the Korea National Health and Nutrition Survey (KNHANES) [5]. Therefore, there has been a lack of comprehensive understanding for assessing household food insecurity in adults. Household food insecurity is a serious public health issue that goes beyond the problem of poverty. An unhealthy diet (malnutrition, nutrition excess, processed food, or micronutrient deficiency) is one of the crucial factors contributing to noncommunicable diseases, which have enormous social and economic costs for individuals, families, and communities [6]. To address this, health authorities emphasize that household food insecurity and the inequalities regarding access to healthy food need to be reduced [7].

Asthma is the most common chronic disorder of the airways, involving a complex interaction of airflow limitation, bronchial hyperresponsiveness, and airway inflammation [8]. Approximately 300 million people worldwide have asthma, and 250,000 deaths are attributable to asthma each year [9]. Traditionally, asthma prevalence is higher among children than adults [10]. However, asthma surveillance data from the Centers for Disease Control and Prevention in 2016 revealed that the prevalence of asthma among children (age < 18 years) and adults (age ≥ 18 years) is the same (8.3% and 8.3%, respectively) [11]. Several studies conducted in Asian countries showed a recent trend of an increasing prevalence of adult asthma [12,13]. Moreover, adults are approximately seven times more likely to die from asthma than children (death rates of 2.8 per million in children and 13.3 per million in adults) [9]. Based on the 2015 Korean guideline for asthma, the prevalence of asthma in adults aged 19 years and older has been steadily increasing from 1.1% in 1998 to 3.1% in 2011 [14]. In particular, the prevalence of asthma after 50 years of age increases again after childhood. Per-capita cost was the highest among patients who were ≥50 years old in Korea [15].

A recent study showed that household food insecurity is significantly associated with asthma in children [16]. The authors suggested the possibility of stress, poor nutrition, and obesity resulting from food insecurity leading to asthma. Meanwhile, another study reported that nutritional outcomes among school-aged children are not tied to household poverty or food insecurity, since the diets of school-aged children might have been supplemented at school [17]. Moreover, recent research has shown that adults in households with children take steps to protect the children against household food shortages by limiting or reducing the quality and quantity of their own meals [18,19]. As a result, adults and elderly individuals may be more susceptible to household food insecurity. The aim of this paper was to determine if there is an association between household food insecurity and the prevalence of asthma in Korean adults by using population-based data.

## 2. Materials and Methods

### 2.1. Study Population

This study was based on data obtained from the Sixth KNHANES, a nationally representative survey conducted by the Korea Centers for Disease Control and Prevention between 2013 and 2015. Detailed information about the KNHANES is summarized on the KNHANES website (http://knhanes.cdc.go.kr). The target population of this survey was noninstitutionalized Korean citizens. To obtain results representative of the entire Korean population, sampling weights indicating the probability of being sampled were assigned to each participant. For the 2013–2015 KNHANES, citizens were informed that they had been randomly selected as a household to voluntarily participate in the nationally representative survey conducted by the Korean Ministry of Health and Welfare. All study participants provided written informed consent. A total of 18,034 participants aged 20 years were included in the KNHANES dataset during 2013–2015. After excluding participants with missing data, 14,770 adults were analyzed in the present study. The Institutional Review Board of Yong-In Severance Hospital approved this study (IRB No: 9-2018-0012).

### 2.2. Data Collection

During the KNHANES, physical examinations and health interviews were performed by trained medical staff according to standardized procedures. The detailed protocol for the 2013–2015 KNHANES is available on the KNHANES website (http://knhanes.cdc.go.kr). Body mass index (BMI) was calculated as the weight in kilograms divided by the square of the height in meters. Information about age, household income, education level, residential area, and occupation was collected through the health interview. Information of health-related behaviors was collected via self-report questionnaires. A dietitian-administrated 24-h recall interview was used to assess the subject’s dietary intake.

### 2.3. Definition of Asthma

Asthma status was determined if the person answered “yes” to one of the following two questions: “Have you ever been diagnosed with asthma by a doctor?” or “Are you currently taking or have you taken treatment for asthma in the past 12 months?”.

### 2.4. Assessment of Household Food Security

Household food insecurity was surveyed using a questionnaire developed by the Korea Centers for Disease Control and Prevention based on the 18-item U.S. Household Food Security/Hunger Survey Module [20]. The questionnaire was completed by a major purchaser from each household and included questions about the dietary habits over the past year. A score of 1 was assigned to positive responses regarding food-insecure conditions and a score of 0 was assigned to all other responses. A sum of the scores was used to categorize the household food security status into one of four groups. A “food-secure household” status was assigned for scores of 0–2, regardless of whether there were children in the household. A “food-insecure household without hunger” status was for households with scores of 3–7 with children or scores of 3–5 without children. A moderate “food-insecure household with hunger” status was for households with children with scores of 8–12 or scores of 6–8 for those without children, and severe “food-insecure household with hunger” was for households with children with scores of 13–18 or scores of 9–10 for those without children.

The number of severe “food-insecure household with hunger” was too small. Therefore, we combined the moderate and severe “food-insecure household with hunger” statuses for analyses.

### 2.5. Covariates

Household income was categorized into four groups: Lowest, medium-lowest, medium-highest, and highest. Education levels were grouped into elementary school graduate or less, middle school graduate, high school graduate, and college graduate or higher. Residential areas were classified as urban or rural according to the participants’ addresses. Occupation was categorized as white collar (including managers, professionals, clerks, service/sales workers, and students) or blue collar (agriculture, forestry, craft and related trade workers, fishery workers, plant and machine operators and assemblers, and simple labor) [21]. A person who was currently smoking and had smoked more than 100 cigarettes during their lifetime was defined as a current smoker. To assess perceived stress, the participants were asked “How much stress do you usually feel?”. The response categories were as follows: Only a little, to some extent, to a great extent, and to a very great extent. The level of perceived stress was categorized into two groups: Low level of stress (only a little and to some extent) and high level of stress (to a great extent and to a very great extent). The proportions of carbohydrate, protein, and fat intake were calculated as percentages of the intake calories for each component/total calorie intake. The intake amounts of vitamins (vitamin A (μg retinol equivalents/day), riboflavin (mg/day), thiamin (mg/day), niacin (mg/day), and vitamin C (mg/day)) were also recorded.

### 2.6. Statistical Analysis

Data are presented as mean ± standard errors or percentages (standard errors). Differences in the demographics, and clinical characteristics of the study population according to household food security status were reported using a weighted one-way analysis of variance (ANOVA) for continuous variables, or weighted chi-square test for categorical variables. To assess the nutritional status according to food insecurity, we also applied weighted one-way ANOVA. The odds ratios (ORs) and 95% confidence intervals (CIs) for the presence of asthma according to household food security status were calculated using multiple logistic regression analyses after adjusting for age, sex, and BMI in Model 1 and additionally adjusting for household income, education, residence area, occupation type, and smoking status in Model 2. All analyses were performed using SPSS 23.0 (IBM Corp., Armonk, NY, USA). All statistical tests were two-sided, and statistical significance was determined at a *p* value of <0.05.

## 3. Results

The demographic and clinical characteristics of the study population are summarized in Table 1. The study population comprised mainly urban households with individuals holding white collar occupations, the vast majority of which reported food security.

The clinical characteristics of the study population according to household food security status are presented in Table 2. Participants in the group with household food security were significantly younger than those in the groups with food insecurity and had significantly higher household incomes and education levels and were more likely to hold white collar occupations but less likely to smoke (all *p* values of <0.001). By contrast, adults from households with food insecurity had a significantly higher level of stress (*p* < 0.001). BMI and area of residence were not significantly different among the groups.

The nutritional intakes according to household food insecurity status are summarized in Table 3. The mean calorie and protein intakes were significantly lower in adults from households with food insecurity than in those that were food-secure (*p* < 0.001); the difference was especially pronounced in adults in households scored as food-insecure with hunger. Vitamin intakes (vitamin A, thiamin [vitamin B1], riboflavin [vitamin B2], niacin, and vitamin C) were also significantly lower in adults from households that were food insecure (all *p* values of <0.01). By contrast, carbohydrate intakes were significantly higher in adults from households that were food insecure with hunger (*p* < 0.001). The intake proportions of fats (total, mono- and polyunsaturated fatty acids, and omega-6 [n-6] and n-3 fatty acids) were lowest in adults from households scored as food insecure with hunger, with higher ratios of n-6 to n-3 fatty acids than in adults of households with food security (*p* < 0.001).

**Figure 1:**
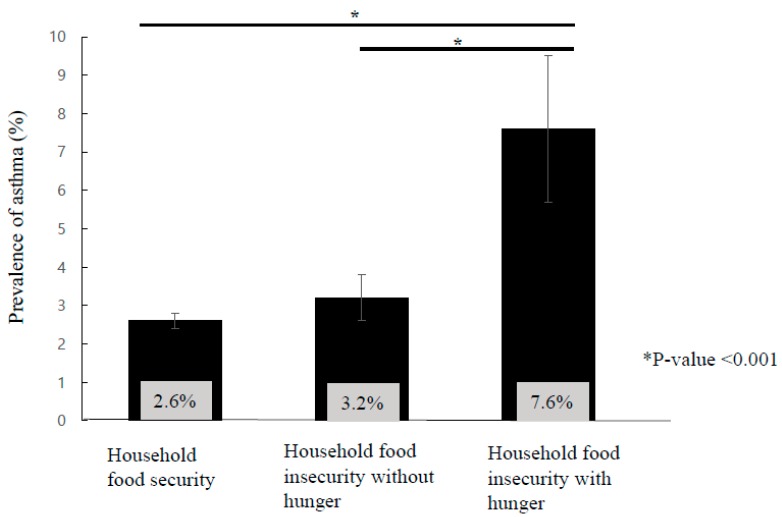
Prevalence of asthma according to household food insecurity. * *p* values were calculated using the general linear model with Bonferroni correction.

Figure 1 shows that the prevalence of asthma was significantly higher in households with food insecurity with hunger than in those without hunger and those with food security (*p* < 0.001).

A multiple logistic regression analysis revealed that household food insecurity was independently associated with asthma after adjusting for possible confounding factors (Table 4), including age, sex, and BMI (Model 1), as well as household income, education level, area of residence, occupation type, and smoking status (Model 2).

**Figure 2:**
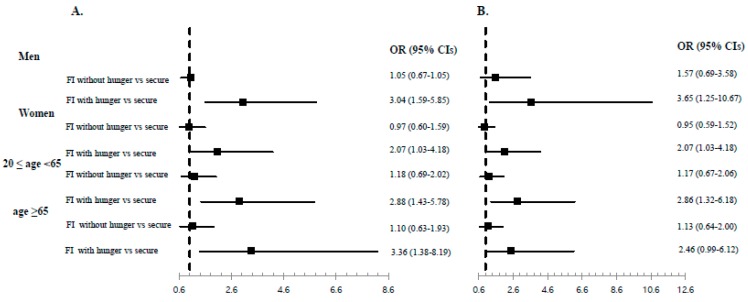
Adjusted ORs and 95% CIs in subgroup analysis by sex and age groups. (**A**) ORs were calculated using multiple logistic analysis after adjusting for age, sex (in age groups), and BMI. (**B**) ORs were calculated using multiple logistic analysis after adjusting for age, sex (in age groups), BMI, education level, household income, smoking, residential area, and occupation type. OR, odds ratio; 95% CI, 95% confidence interval; FI, food insecurity.

Figure 2 presents the forest plots (presented as ORs with 95% CIs) for subgroup analyses of asthma prevalence according to sex and age after adjusting for age, sex, and BMI (Figure 2A) and after adjusting for age, sex, BMI, education level, household income, smoking, residential area, and occupation (Figure 2B). Similar associations between food insecurity with hunger and asthma prevalence remained among men, women, adults (aged 20–65 years), and elderly adults (≥65 years) after adjusting for all confounding factors.

## 4. Discussion

We found that household food insecurity was closely associated with asthma prevalence in a representative sample of Korean adults. Notably, asthma prevalence was significantly higher among adults from households with food insecurity with hunger. The significant association between household food insecurity and asthma remained in subgroup analyses for men, women, adults aged 20–65 years, and elderly adults (≥65 years).

Several studies, including those of cross-sectional and longitudinal designs, have demonstrated that food insecurity is associated with a higher prevalence of asthma in school-aged children [16,22,23]. Children between 5 and 17 years of age are known to have the highest asthma prevalence rates [24]. Although it is difficult to estimate the global asthma prevalence due to a lack of standardized measures among surveys [25], recent studies have shown that childhood asthma prevalence rates increased until 2009 and have plateaued or declined since 2010 [26,27]. The heterogeneity of asthma prevalence among adults also makes estimates of its prevalence difficult [28]. However, data from the 2016 National Health Interview Survey indicate that the asthma prevalence among adults is not decreasing [29]. Although the prevalence of asthma in Asia is lower than in Western countries [30], the prevalence increases with age among Asian adults [12,24]. Additionally, the etiologies of asthma differ between children and adults [31,32]. Adult asthma is less often associated with genetic predisposition [33] and is more affected by environmental triggers, hormonal factors, respiratory infection, and stress [32]. Adult asthma patients have a poorer prognosis [34], worse lung function [35], and higher mortality [10] than children with asthma.

With regard to household food insecurity, adults and elderly individuals may be the first to experience the effects, as they reduce their meals to protect the children in the household from food insufficiencies [18,19]. Despite the greater severity of asthma effects in adults and their vulnerability to food insecurity, this is, to the best of our knowledge, the first report on the association between household food insecurity and asthma in adults.

Several mechanisms might explain this significant association. First, an unhealthy diet resulting from household food insecurity may influence systemic inflammation that contributes to asthma [36]. Many observational studies have shown that food insecurity is associated with a poor-quality diet, including lower intakes of fruits, vegetables, protein, and micronutrients than in diets from food-secure households [17,19,37,38]. Similar to previous research [38], adults of households with food insecurity with hunger in the present study had significantly lower intakes of total energy and lower percentages of protein, fat, and vitamin intakes. Fruit, vegetables, and their antioxidants might reduce airway inflammation [36]. For example, Wood and colleagues [39] found that tomato intake in asthmatic adults reduced airway neutrophil influx and neutrophil elastase in sputum. In our study, adults from households with food insecurity with hunger had higher carbohydrate intakes and n-6-to-n-3 fatty acid ratios than adults from households that were food secure. The higher carbohydrate intake might suggest that subjects with food insecurity or low incomes chose energy-dense foods containing large amounts of sugar and refined grains to reduce cost [40]. A high consumption of sugar is associated with allergic inflammation of the airways in mice [41]. A higher dietary n-6-to-n-3 ratio has been suggested to contribute to asthma by increasing inflammatory cells and the synthesis of prostaglandin E2 [42].

Stress may be a second mechanism to explain the association between household food insecurity and asthma in adults. Food insecurity might act as a chronic stressor and subsequently lead to negative health outcomes [37]. Stress contributes to asthma by affecting proinflammatory processes [43]. A longitudinal Copenhagen City Heart Study demonstrated that a high level of stress is associated with higher asthma incidences than a low level of stress (OR, 2.32; 95% CI: 1.47–3.65) [39]. In the present study, the proportion of participants with a high level of stress was significantly higher in households with food insecurity with hunger.

As a third mechanism, unhealthy behaviors, such as smoking, physical inactivity, and poor chronic disease management, may influence the poor health outcomes in individuals with household food insecurity [44]. Environmental, psychosocial, behavioral, and lifestyle risk factors have complex interactions with asthma and asthma management [45].

Our study has several limitations. First, we could not assess causality due to the cross-sectional design. Although we hypothesized that household food insecurity contributes to asthma onset, it is not clear that the asthma preceded the exposure to food insecurity or how the length of the exposure contributed to the asthma. Second, the prevalence of asthma was defined according to the participants’ responses on the survey rather than standardized measures. Third, household food insecurity might have been underestimated. There is a possibility that households with hunger refused to participate in the interview or did not report their difficult situation. Finally, since we used KNHANES data, our results do not reflect all ethnic groups and individuals from other countries. Nevertheless, a strength of this study is that household food insecurity was assessed using validated measures in a large population-based study. Another strength is that this is the first study to establish the association between household food insecurity and asthma in adults.

## 5. Conclusions

Household food insecurity with hunger is closely associated with asthma prevalence in Korean adults. Inequalities in the accessibility, sufficiency, and availability of foods might lead to chronic diseases such as asthma. More intense and active strategies to screen for food-insecure households and to prevent food inequalities are required to reduce the prevalence of asthma.

## Figures and Tables

**Table 1:** Subject characteristics.

Characteristic	Value *
No. of subjects (unweighted)	14,770
Age (years)	46.8 ± 0.2
Sex	
Male	49.5 (0.4)
Female	50.5 (0.4)
BMI (kg/m²)	23.8 ± 0.0
Household income (percentile)	
Lowest	15.5 (0.6)
Medium-lowest	24.6 (0.7)
Medium-highest	29.2 (0.7)
Highest	30.7 (0.9)
Education level	
Elementary school or less	16.7 (0.5)
Middle school	9.2 (0.3)
High school	36.9 (0.6)
College or higher	37.2 (0.8)
Residential area	
Urban	82.7 (1.5)
Rural	17.3 (1.5)
Occupation type	
White collar	76.8 (0.6)
Blue collar	23.2 (0.6)
Current smoker	
Yes	22.5 (0.5)
No	77.8 (0.5)
Perceived stress	
High	26.0 (0.5)
Low	74.0 (0.5)
Food insecurity	
Secure	91.8 (0.4)
Insecure without hunger	6.8 (0.4)
Insecure with hunger	1.3 (0.1)

* Data are mean ± standard error or percentage (standard error).

**Table 2:** Clinical characteristics according to the household food security status in Korean adults.

Characteristic	Household Food Security Status *			*p* Value
	Food Secure	Food Insecure		
		Without Hunger	With Hunger	
No. of subjects (unweighted)	13,508	1052	210	
Age (years)	46.5 ± 0.2	48.9 ± 0.7	51.1 ± 1.5	<0.001
Sex				0.018
Male	49.8 (0.4)	45.1 (1.7)	50.2 (3.5)	
Female	50.2 (0.4)	54.9 (1.7)	49.8 (3.5)	
BMI (kg/m²)	23.8 ± 0.0	24.03 ± 0.2	23.71 ± 0.3	0.346
Household income percentile				<0.001
Lowest	13.3 (0.5)	35.7 (2.4)	57.0 (5.5)	
Medium-lowest	23.4 (0.7)	39.4 (2.5)	26.3 (5.0)	
Medium-highest	30.2 (0.7)	19.5 (2.0)	15.4 (4.6)	
Highest	33.2 (0.9)	5.3 (1.3)	1.4 (1.3)	
Education level				<0.001
Elementary school or less	15.4 (0.5)	29.5 (1.8)	38.9 (4.8)	
Middle school	8.9 (0.3)	11.8 (1.2)	18.2 (3.7)	
High school	36.8 (0.7)	39.7 (2.1)	27.4 (4.6)	
College or higher	39.0 (0.8)	19.0 (1.8)	15.6 (4.1)	
Residential area				0.844
Urban	82.7 (1.5)	82.7 (2.4)	84.9 (3.4)	
Rural	17.3 (1.5)	17.3 (2.4)	15.1 (3.4)	
Occupation type				
Blue collar	22.6 (0.6)	29.9 (1.8)	26.6 (4.0)	<0.001
White collar	77.4 (0.6)	70.1 (1.8)	73.4 (4.0)	
Perceived stress				
High	25.0 (0.5)	35.2 (1.8)	48.3 (4.2)	<0.001
Low	75.0 (0.5)	54.8 (1.8)	51.7 (4.2)	

* Data are mean ± standard error or percentage (standard error).

**Table 3:** Nutrition intake status according to household food security status in Korean adults.

Category *	Household Food Security Status ^†^			*p* Value
	Food Secure	Without Hunger	With Hunger	
Total calories (kcal/day)	2124.9 ± 11.9	1991.8 ± 44.3	1606.9 ± 77.8	<0.001
Carbohydrate (%)	62.6 ± 0.2	64.9 ± 0.5	66.5 ± 1.5	<0.001
Protein (%)	13.8 ± 0.0	13.2 ± 0.2	12.4 ± 0.4	<0.001
Fat (%)	19.1 ± 0.1	17.6 ± 0.4	15.5 ± 0.9	<0.001
MUFA (%)	6.0 ± 0.0	5.4 ± 0.1	4.5 ± 0.3	<0.001
PUFA (%)	4.8 ± 0.0	4.3 ±0.1	4.1 ±0.3	<0.001
n-6 (%)	4.1 ± 0.0	3.8 ± 0.1	3.5 ± 0.3	0.001
n-3 (%)	0.71 ± 0.0	0.62 ± 0.0.	0.59 ± 0.0	<0.001
n-6/n-3 ratio	7.9 ± 0.1	8.5 ± 0.2	10.1 ± 0.9	0.004
Vitamin A (μg RE/day)	772.2 ± 12.0	700.6 ± 35.7	569.0 ± 69.69	0.004
Vitamin B1 (mg/day)	2.1 ± 0.0	1.9 ± 0.0	1.5 ± 0.1	<0.001
Vitamin B2 (mg/day)	1.4 ± 0.0	1.3 ± 0.0	1.0 ± 0.1	<0.001
Niacin (mg/day)	17.2 ± 0.1	15.1 ± 0.4	12.0 ± 0.9	<0.001
Vitamin C (mg/day)	103.8 ± 1.8	90.8 ± 5.0	55.8 ± 5.7	<0.001

* Percentages are intake (g) × 4 kcal (in case of carbohydrate and protein) or 9 kcal (in case of fats)/total calorie intake × 100; MUFA, monounsaturated fatty acid; PUFA, polyunsaturated fatty acid; n-6, omega-6; n-3, omega-3; RE, retinol equivalents. ^†^ Data are mean ± standard errors.

**Table 4:** Odds ratios (ORs) (95% CIs) for asthma according to household food security status in Korean adults.

Model *	Household Food Security Status		
	Food Secure	Food Insecure	
		Without Hunger	With Hunger
Unadjusted	1.00	1.26 (0.83–1.90)	3.12 (1.81–5.36)
1	1.00	1.19 (0.78–1.81)	3.05 (1.78–5.26)
2	1.00	1.12 (0.73–1.74)	2.44 (1.33–4.46)

* Model 1, adjusted for age, sex, and body mass index (BMI); Model 2, adjusted for age, sex, BMI, education level, household income, smoking, residential area, and occupation type.
